# Efficacy of intravaginal electrical stimulation with different treatment frequency in women with refractory idiopathic overactive bladder

**DOI:** 10.1590/S1677-5538.IBJU.2021.0837

**Published:** 2022-03-30

**Authors:** Necmettin Yildiz, Hakan Alkan, Gulin Findikoglu

**Affiliations:** 1 Department of Physical Medicine and Rehabilitation Pamukkale University Faculty of Medicine Denizli Turkey Department of Physical Medicine and Rehabilitation, Pamukkale University Faculty of Medicine, Denizli, Turkey

**Keywords:** Urinary Bladder, Overactive, Therapeutics, Urinary Incontinence

## Abstract

**Objective:**

The aim of this study is to evaluate the effect of intravaginal electrical stimulation (IVES) therapies with different treatment frequencies (two or five days in a week) added to bladder training (BT) on incontinence-related quality of life (QoL) and clinical parameters in women with refractory idiopathic overactive bladder (OAB).

**Material and Methods:**

Fifty-two women with refractory idiopathic OAB were randomized into two groups as follows: Group 1 (n:26) received BT and IVES, two times in a week, for 10 weeks and Group 2 (n:26) received BT and IVES five times in a week, for 4 weeks. IVES was performed 20 minutes in a day, a total of 20 sessions for both groups. Women were evaluated for incontinence severity (24h pad test), pelvic floor muscles strength (perineometer), 3-day voiding diary (frequency of voiding, nocturia, incontinence episodes, and the number of pads), symptom severity (OAB-V8), quality of life (IIQ-7), treatment success (positive response rate), cure/improvement rate and treatment satisfaction (Likert scale).

**Results:**

There was no statistically significant differences in all parameters between the two groups at the end of the treatment. It was found that the treatment satisfaction scores, cure/improvement and positive response rates were not significantly different between two groups (p>0.05).

**Conclusion:**

We concluded that the application of IVES twice a week or 5 times a week added to BT were both effective on incontinence-related QoL and clinical parameters in women with refractory idiopathic OAB. These two IVES frequencies had similar clinical efficacy and patient satisfaction with a slight difference between them; 5 times per week IVES has a shorter treatment duration.

## INTRODUCTION

Overactive bladder (OAB) is a symptom complex defined as urgency, with or without urgency urinary incontinence (UUI), usually with frequency and nocturia in the absence of urinary tract infection ( 1 ). Currently, a wide range of therapeutic options exists for the treatment of OAB.

Electrical stimulation (ES) is one of the techniques used in urogynecological physiotherapy, which uses implanted or non-implanted electrodes ( 2 ). Intravaginal ES (IVES) is a conservative treatment option used in patients with OAB and UUI for detrusor inhibition. It has been suggested that IVES probably targets the detrusor muscle or pelvic floor muscle (PFM) or afferent innervation in UUI. According to the European Association Urology Guidelines, ES may improve urinary incontinence compared to sham treatment in adults with urinary incontinence ( 3 ). The duration of IVES programs varied from 4 weeks to 6 months in women with idiopathic OAB in the literature, although IVES was applied for 4-12 weeks commonly in practice ( 4 - 11 ). In most studies, IVES was applied 2-3 times a week ( 4 - 11 ), whereas it was applied more frequently in fewer studies ( 12 - 14 ). Despite that, no randomized study compared the different IVES treatment frequencies in women with idiopathic OAB, and thus, there is no evidence for which frequency of treatment is the most effective one. It should be kept in mind that the different stimulation frequencies may lead to different results. Some studies evaluating the efficacy of IVES included subjects were not used antimuscarinics within the last 4 week or antimuscarinic-naive patients with OAB ( 4 , 15 ), while some included patients with OAB who were unresponsive or intolerant to antimuscarinics ( 5 , 16 ). As a result, IVES appears to be a non-invasive and effective therapy used both as first-line treatment, as well as in managing of refractory patients with idiopathic OAB.

Our study is the first prospective randomized trial that compares the efficacy of IVES with different treatment frequencies in women with refractory idiopathic OAB. In this study, we aimed to assess the effect of IVES applied for 2 times vs 5 times in a week added to bladder training (BT) on quality of life (QoL) and the clinical parameters associated with idiopathic OAB. The results of our study will be of great benefit in determining the effectiveness of different treatment frequencies of IVES in women with idiopathic OAB. Thus, more effective treatment frequency of IVES (2 or 5 times in a week) can be determined or if they are of similar effectiveness, the frequency and duration of treatment may be left to the choice of the patients and the physicians taking into account non-treatment conditions.

## MATERIAL AND METHODS

This study was planned as a prospective, randomized clinical trial. The trial was carried out in the Urogynecological Rehabilitation Unit of Physical Medicine and Rehabilitation Department, between February 2021 and August 2021. The local ethics committee approved the study (E-60116787-020-4274). This study was registered with ClinicalTrials.gov number, NCT04734301. All women were informed about the purpose and contents of the study and all women signed written consent to participate in the study.

Considering a 50% or greater improvement in incontinence episodes in the previously study, the optimum sample size should be 26 cases in each arm (a total of 52 women) with a level of significance of 95% (α=5%), a power of 95% (ß=0.05) ( 4 ). Sample size calculation was performed by the physician who was blinded to groups using G*Power 3.1 Statistical Power Analysis for Microsoft Windows and Mac. Statistics.

We recruited 74 women with complaints of OAB who were referred to the Urogynecological Rehabilitation Unit and other related outpatient clinics. Women over the age of 18 with the clinical diagnosis of idiopathic OAB, and who were intolerant or unresponsive to antimuscarinics and discontinued at least 4 weeks ago, and who were able to give written informed consent and understand the procedures were included in this study. The criteria for exclusion were as follows: women who had stress urinary incontinence; a history of conservative therapy (BT, ES) for OAB within 6 months; urogynecological surgery within 3 months; current vulvovaginitis or urinary tract infections or malignancy; pregnancy; cardiac pacemaker or implanted defibrillator; anatomic structural disorders of the genital region that did not allow to apply the vaginal probe; the strength of PFM less than 3/5 (graded as modified Oxford scale, min:0-max:5); the pelvic organ prolapse quantification (POP-Q) (stage 2 or more); neurogenic bladder; the peripheral or central neurologic pathology; ultrasonographic evidence of post-void residual urine volume more than 100 mL (using Telemed Micrus portable ultrasonography (the Lithuania) device ( 17 ), and allergy to condom or lubricant gel that is used with perineometer/vaginal probe were excluded.

**Figure 1:**
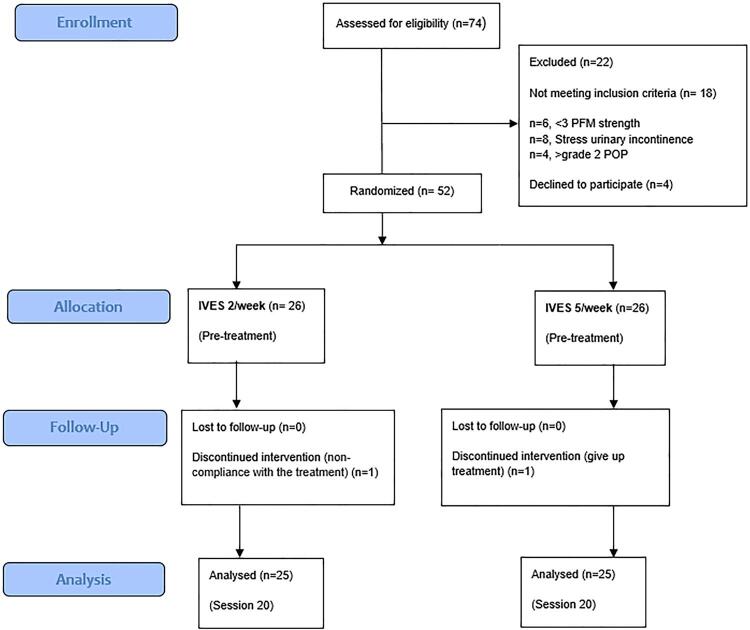
CONSORT participant flow diagram for randomized, controlled trials of non pharmacologic treatment.

Seventy-four women with idiopathic OAB were recruited for eligibility and fifty-two of them who fulfilled the inclusion/exclusion criteria were included for this study. The flow chart is shown in Figure-1 . By using a random number generator, 52 women were randomized into two groups as follows: Group 1 received BT+IVES (2 times in a week) (n:26), Group 2 received BT+IVES (5 times in a week) (n:26) ( Figure-1 ). A random allocation sequence was generated at a 1:1 ratio.

**IVES** = Intravaginal electrical stimulation; **PFM** = pelvic floor muscle; **POP** = pelvic organ prolapse

### Bladder Training (BT)

All women were informed about BT that consisted of four stages and lasted for 30 minutes. Then, it was given as a written brochure to be implemented as a home program. At the first stage, the women were familiarized with the location of the PFM and the pelvic anatomy and pathophysiology. After that information session, squeezing the PFM was shown in practice at least once to use in the urgency suppression strategies via digital palpation technique. The second stage including urgency suppression strategies was aimed to delay urination, inhibit detrusor contraction, and prevent urgency by squeezing the PFM several times on a row, breathing deeply, giving their attention to another job for a while, and self-motivating. In the third stage, a timed voiding program was started. It was carried out in 2 steps: a timed voiding and increasing the time between urination considering the voiding diary. At the last stage, the women were encouraged to continue BT ( 4 , 5 , 18 , 19 ).

### Group 1: Two times IVES in a week (2/week IVES)

IVES was applied in addition to BT in this group. IVES was performed in lithotomy position via a stimulation device (Enraf Nonius Myomed 632) with a vaginal probe. IVES was performed two days a week, a total of 20 sessions for 10 weeks. Every session lasted 20 minutes. The stimulation parameters were a 10 Hz of frequency, a 5-10 s of work-rest cycle duration and, a 100 ms of pulse width. The symmetric biphasic pulse wave could be delivered over a range of 1-100 mA (with respect to the patient’s discomfort level feedback) ( 4 , 5 , 11 , 20 ).

### Group 2: Five times IVES in a week (5/week IVES)

This group was also treated with the IVES in addition to all components of the BT in Group 1. IVES was performed in the same way as Group 1, except for the frequency and the total duration of treatment. IVES was performed five days a week, a total of 20 sessions for 4 weeks. Every session lasted 20 minutes. Stimulation parameters were the same as Group 1 ( 4 , 5 , 11 , 20 ).

IVES sessions were performed by an experienced urogynecological rehabilitation nurse in all groups. During the treatment, all women were advised to continue the medical treatment which was not related to incontinence. Participants were asked to fill in a one-day bladder diary once every 5 sessions to continue the timed voiding program, which is part of BT in both groups. Compliance with the BT was achieved with the daily checklist during 20 sessions and the bladder diaries of women were checked every 5 sessions to rearrange the timed voiding program. Women who did not fill in more than 20% of the daily checklist and women who missed any therapy sessions for two groups were excluded from the study ( 5 , 8 ).

### Evaluation Parameters

The primary outcome measure was accepted as the improvement in incontinence episodes (positive response rate), according to literatüre ( 14 , 21 ). To determine positive response rate, reduction in incontinence episodes was collected from the 3-day bladder diary. Women with ≥ a 50% reduction in incontinence episodes were considered positive responders ( 4 , 22 ). Furthermore, the severity of incontinence, PFM strength, symptom severity, frequency of voiding, nocturia, number of pads as well as QoL were secondary outcome measures. The 24-hour pad test was carried out to evaluate the severity of incontinence ( 23 ). PFM strength was evaluated with Peritron 9300 device ( 24 ). Overactive Bladder Questionnaire (OAB-V8) was used to evaluate the symptom severity in patients with OAB in this study. The OAB-V8 consists of 8 questions in which the patients can be classified with respect to the symptom severity: none (0), very little ( 1 ), a little ( 2 ), quite a few ( 3 ), very ( 4 ), and too many ( 5 ). The total score ranges from 0-40 ( 25 , 26 ). The frequencies of voiding, nocturia, and the number of pads used were collected from the 3-day bladder diary. The Quality of Life-Incontinence Impact Questionnaire (IIQ7) was used to assess specific QoL related to incontinence ( 27 ). In addition, cure-improvement rate and treatment satisfaction were evaluated. In a 24-hour pad test, amount of urine that was under 1.3 gr was considered as a cure. The improvement rate was assessed in terms of 50% and more reduction in wet weight compared to baseline measurements in the 24-hour pad test ( 23 ). Women evaluated the change in their urinary incontinence on a 5-point Likert scale (5, very satisfied; 1, very unsatisfied) ( 4 , 5 ). All the evaluation tests were performed by another physician who was blinded to the groups in the initial visit and at the end of the treatment (20^th^ session), except for the positive response rate, cure/improvement rate, and the treatment satisfaction parameters which were evaluated only at the 20^th^ sessions.

### Statistics

SPSS17.0 software (SPSS, Chicago, IL) was used for the statistical analysis. In each group, measurable parameters were tested with the Kolmogorov-Smirnov test for the evaluation of normal distribution. Because the distributions were not normal, non-parametric tests were used in the statistical evaluation. Mann-Whitney U-test and χ2 test were used for inter-group comparisons. Wilcoxon tests were used for intra-group comparison of parameters at different point of times. P<0.05 was accepted as statistically significant.

## RESULTS

One woman was withdrawn because of doing BT irregularly in Group 1 and one woman gave up treatment in Group 2. The data of drop outs were excluded from the study ( Figure-1 ).

The demographic data at the beginning was shown in Table-1 . There were no statistically significant differences in the demographic data. Table-2 shows the comparison of the assessment parameters at the baseline and the end of the treatment (20^th^ session) for each group. Both groups were not significantly different for the severity of incontinence, PFM strength, frequency of voiding, incontinence episodes, nocturia, number of pads, symptom severity, and QoL parameters at baseline (p>0.05) ( Table-2 ).

**Table 1:** Demographic data of women with idiopathic overactive bladder.

	Group 1 n:25	Group 2 n:25	P^1^	P^2^
Age (year) (mean±SD)	56.64±10.02	58.72±14.20	0.560	
Height (cm) (mean±SD)	158.76±6.12	158.68±5.72	0.899	
Weight (kg) (mean±SD)	75.00±12.76	76.52±10.68	0.907	
BMI (kg/m^2^) (mean±SD)	29.82±5.16	30.39±4.27	0.816	
Duration of incontinence (month) (mean±SD)	81.60±67.79	79.68±82.16	0.640	
**Education, n(%)**				
Primary	18(72)	19(76)		
High school	4(16)	3(12)		
>High school	3(12)	3(12)		0.952
**Smoking, n(%)**				
No	20 (80)	18(72)		
Yes	5(20)	7(28)		0.293
**Cup of tea/day, n(%)**				
1-2 cup	9(36)	7(28)		
≥3 cup	16(64)	18(72)		0.424
**Cup of coffee/day, n(%)**				
No	12(48)	12(48)		
1-2 cup	12(48)	12(48)		
≥3 cup	1(4)	1(4)		1.000
**Alcohol intake, n(%)**				
No	25(100)	25(100)		
Yes	0(0)	0(0)		1.000
**Delivery, n(%)**				
No	1(4)	2(8)		
1-3	17(68)	16(64)		
≥4	7(28)	7(28)		0.794
**Delivery type, n(%)**				
No	1(4)	2(8)		
NSVD	23(92)	20(80)		
Sectio	1(4)	3(12)		0.363
**Episiotomy, n(%)**				
No	16(64)	13(52)		
Yes	9(36)	12(48)		0.416
**Menopausal status, n(%)**				
Premenopause	7(28)	8(32)		
Postmenopause	18(72)	17(68)		0.758
**HRT use, n(%)**				
No	24(96)	20(80)		
Yes	1(4)	5(20)		0.082

**Group 1** - Two times in a week intravaginal electrical stimulation (2/week IVES); **Group 2** - Five times in a week intravaginal electrical stimulation (5/week IVES); **HRT** , Hormone replacement therapy; **BMI** = Body mass index; **NSVD** = normal spontaneous vaginal delivery; **P1** = Mann-Whitney U-test; **P2** = Pearson χ2 test.

**Table 2:** Comparison of groups with respect to evaluation parameters.

	Group 1 n:25 (mean±SD)	Group 2 n:25 (mean±SD)	Mann-Whitney-U test p
**Severity of incontinence - 24h Pad test (gr)**			
Pretreatment	40.22±22.76	43.24±39.23	0.614
Session 20	7.60±9.88 *	9.84±15.19 *	0.899
**PFM strength - Perineometer (cmH**_**2**_ **O)**			
Pretreatment	22.72±10.74	20.76±11.79	0.559
Session 20	27.44±13.25 *	24.92±11.78 *	0.697
**Bladder diary**			
**a. Frequency**			
Pretreatment	11.64±3.63	10.92±4.22	0.232
Session 20	6.24±1.69 *	6.40±1.93 *	0.819
**b. Nocturia**			
Pretreatment	2.68±2.21	2.80±1.77	0.599
Session 20	1.00±0.91 *	0.84±0.98 *	0.433
**c. Incontinence episodes**			
Pretreatment	4.12±2.86	5.20±4.78	0.492
Session 20	0.68±1.14 *	1.00±1.29 *	0.268
**d. Number of pads**			
Pretreatment	3.40±2.19	3.00±1.97	0.538
Session 20	1.56±1.44 *	0.88±0.88 *	0.087
**Symptom severity - OAB-V8**			
Pretreatment	26.12±5.20	27.84±7.39	0.484
Session 20	8.28±4.56 *	8.88±7.38 *	0.861
**Quality of life - IIQ7**			
Pretreatment	14.12±5.73	14.60±5.84	0.719
Session 20	6.20±6.05 *	6.00±7.27 *	0.604
**Treatment satisfaction ( 1 - 5 )**			
Session 20	4.48±0.71	4.40±0.81	0.825

**Group 1** - Two times in a week intravaginal electrical stimulation (2/week IVES); **Group 2** - Five times in a week intravaginal electrical stimulation (5/week IVES); **OAB-V8** = Overactive Bladder Questionnaire; **IIQ-7** = Incontinence Impact Questionnaire; **PFM** = Pelvic floor muscle; * = P<0.05: Wilcoxon test compare with baseline values

Statistically significant improvements were found in the severity of incontinence, PFM strength, frequency of voiding, incontinence episodes, nocturia, number of pads, symptom severity, and QoL parameters for the two groups at the end of the treatment (20th session) compared to the baseline values (p<0.05). There was no statistically significant differences in all parameters between the two groups at the end of the treatment. Moreover, it was observed that the treatment satisfaction scores were similar in both groups (p>0.05) ( Table-2 ). Similar values were found between Groups 1 and 2 in both positive response and cure/improvement rates (p=0.193 and p=0.637, respectively). Positive response rates in Group 1 and Group 2 were 88% and 92%, respectively. The cure and improvement rates were 44% and 88% in Group 1, while they were 52% and 92% respectively in Group 2.

No serious adverse events were reported in both groups except temporary discomfort due to vaginal irritation in two women in each group.

## DISCUSSION

In this prospective, randomized clinical trial, we have investigated the effectiveness of both “twice a week” and “5 times a week” IVES treatment added to BT for a total of 20 sessions on QoL and clinical parameters associated with incontinence in women with refractory idiopathic OAB. As a result, we have observed significant improvements in terms of incontinence severity, PFM strength, frequency of voiding, incontinence episodes, nocturia, number of pads, symptom severity, and QoL at the 20th session evaluations in both groups when compared with baseline. There was no significant difference between “twice a week IVES” and “5 times a week IVES” groups in all parameters. It was observed that the treatment satisfaction scores, cure/improvement, and positive response rates were similar in both groups.

There was no randomized study that compared different electrical current parameters or different treatment frequencies and thus, there was no evidence of which parameters or treatment frequencies were the most effective ones. In this context, our study is the first study to compare the efficacy of different IVES treatment frequencies in women with idiopathic OAB. Our findings indicated that these two IVES frequencies (twice a week and 5 times a week) had similar clinical efficacy and patient satisfaction. The most commonly reported electrical current frequency by the authors was 10 Hz for OAB. Working and resting times of the current ranged from 2 sn to 10 sn in the literature, and the most commonly used ones were 5 sn and 10 sn, respectively. All authors who described the intensity of electrical current used the maximum intensity depending on the patient’s tolerance (max 100 mA). In most cases, the application time used was 20 minutes ( 2 , 4 , 5 ). In our study, the most frequently used electrical current parameters and application time were used in accordance with the literature ( 2 , 4 , 5 ). However, better methodological quality studies are needed to know the optimal current modality and parameters for OAB.

Up to our knowledge, there are only three studies including BT+IVES treatment arm in women with idiopathic OAB in the literature ( 4 , 5 , 19 ). In the first of these studies, BT+IVES was not found to be effective compared to BT alone. Women received relatively few treatment sessions (once a week, 9 sessions), besides the improvement and positive response rates were not mentioned in this study ( 19 ). Two recent randomized controlled studies reported that BT+IVES was more effective than BT alone, when IVES was applied to women 3 times a week for a total of 24 sessions ( 4 , 5 ). These studies used the improvement rate which was determined according to the 24-hour pad test results, and positive response rate which was calculated from the ≥50% reduction in incontinence episodes in accordance with our study. The improvement rates (82.4% and 89.7%, respectively), and positive response rates (88.2% and 86.2%, respectively) of these studies were similar to our study ( 4 , 5 ). However, it should be taken into account that the frequency of IVES applied in each group was different from these studies in our study. It should be kept in mind that different treatment frequencies other than these may lead to different results. We think that this issue is still open for research.

It has been reported that a minority of women developed adverse effects such as pain, discomfort, hypersensitivity, irritation, tingling in the thigh, hemorrhage, diarrhea, bladder spasm, and vaginal or urinary infection related to IVES ( 2 ). In general, IVES was well tolerated by women except for temporary discomfort due to vaginal irritation in two women in each group in our study.

The scientific and clinical importance of our study results are as follows: (i) This is the first randomized clinical trial to evaluate the efficacy of IVES at different treatment frequencies in women with idiopathic OAB; (ii) Clinical efficacy is similar for “twice a week IVES” and “5 times a week IVES” treatments added to BT; (iii) The results of our study will be of great benefit in preferring the treatment frequency (two or five times in a week) and thus the treatment duration (10 or 4 weeks) of IVES for the women with idiopathic OAB and their physicians.

There are some limitations in our study. One of the limitations of this study was that there was no data about the long-term follow-up of the patients. Another limitation was that there was no data about urodynamics. The lack of an isolated BT group makes it impossible to rule out the possibility of an isolated BT effect on the result with potentially null action for IVES in women with idiopathic OAB. In addition, when interpreting our study results, it should be taken into account that the BT program takes longer in women who received IVES twice a week compared to women who received IVES 5 times a week (10 weeks and 4 weeks respectively).

## CONCLUSIONS

We concluded that both the twice-a-week IVES and the 5 times a week IVES added to BT were effective on both incontinence-related QoL and clinical parameters in women with refractory idiopathic OAB. These two IVES frequencies had similar clinical efficacy and patient satisfaction with a slight difference between them; 5 times per week IVES has a shorter duration of treatment. It will be of great benefit in preferring the treatment frequency or treatment duration for the women with idiopathic OAB and their physicians.

## ABBREVIATIONS

BT: Bladder Training
ES: Electrical Stimulation
IVES: Intravaginal Electrical Stimulation
OAB: Overactive Bladder
PFM: Pelvic Floor Muscle (PFM)
QoL: Quality of Life
UUI: Urgency Urinary Incontinence

## References

[1] Bo K, Frawley HC, Haylen BT, Abramov Y, Almeida FG, Berghmans B, et al. An International Urogynecological Association (IUGA)/International Continence Society (ICS) joint report on the terminology for the conservative and nonpharmacological management of female pelvic floor dysfunction. Int Urogynecol J. 2017;28:191-213. 10.1007/s00192-016-3123-4 27921161

[2] Jerez-Roig J, Souza DL, Espelt A, Costa-Marín M, Belda-Molina AM. Pelvic floor electrostimulation in women with urinary incontinence and/or overactive bladder syndrome: a systematic review. Actas Urol Esp. 2013;37:429-44. English, Spanish. 10.1016/j.acuro.2012.08.003 23246103

[3] Burkhard FC, Bosch JLHR, Cruz F, Lemack GE, Nambiar AK, Thiruchelvam N, et al. The European Association of Urology (EAU) Guidelines. EAU Guidelines on urinary incontinence in adults. In: EAU Guidelines, 2019. [Internet]. Available at: <http://uroweb.org/guideline/urinary-incontinence/>

[4] Firinci S, Yildiz N, Alkan H, Aybek Z. Which combination is most effective in women with idiopathic overactive bladder, including bladder training, biofeedback, and electrical stimulation? A prospective randomized controlled trial. Neurourol Urodyn. 2020;39:2498-508. 10.1002/nau.24522 32960999

[5] Yildiz N, Alkan H, Sarsan A. Efficacy of intravaginal electrical stimulation added to bladder training in women with idiopathic overactive bladder: A prospective randomized controlled trial. Int Braz J Urol. 2021;47:1150-9. 10.1590/S1677-5538.IBJU.2021.0161 PMC8486445 34469668

[6] Ozdedeli S, Karapolat H, Akkoc Y. Comparison of intravaginal electrical stimulation and trospium hydrochloride in women with overactive bladder syndrome: a randomized controlled study. Clin Rehabil. 2010;24:342-51. 10.1177/0269215509346092 20212061

[7] Wang AC, Chih SY, Chen MC. Comparison of electric stimulation and oxybutynin chloride in management of overactive bladder with special reference to urinary urgency: a randomized placebo-controlled trial. Urology. 2006;68:999-1004. 10.1016/j.urology.2006.05.038 17113893

[8] Barroso JC, Ramos JG, Martins-Costa S, Sanches PR, Muller AF. Transvaginal electrical stimulation in the treatment of urinary incontinence. BJU Int. 2004;93:319-23. 10.1111/j.1464-410x.2004.04608.x 14764129

[9] Spruijt J, Vierhout M, Verstraeten R, Janssens J, Burger C. Vaginal electrical stimulation of the pelvic floor: a randomized feasibility study in urinary incontinent elderly women. Acta Obstet Gynecol Scand. 2003;82:1043-8. 10.1034/j.1600-0412.2003.00130.x 14616279

[10] Arruda RM, Castro RA, Sousa GC, Sartori MG, Baracat EC, Girão MJ. Prospective randomized comparison of oxybutynin, functional electrostimulation, and pelvic floor training for treatment of detrusor overactivity in women. Int Urogynecol J Pelvic Floor Dysfunct. 2008;19:1055-61. 10.1007/s00192-008-0586-y 18330483

[11] Wang AC, Wang YY, Chen MC. Single-blind, randomized trial of pelvic floor muscle training, biofeedback-assisted pelvic floor muscle training, and electrical stimulation in the management of overactive bladder. Urology. 2004;63:61-6. 10.1016/j.urology.2003.08.047 14751349

[12] Brubaker L, Benson JT, Bent A, Clark A, Shott S. Transvaginal electrical stimulation for female urinary incontinence. Am J Obstet Gynecol. 1997;177:536-40. 10.1016/s0002-9378(97)70142-x 9322620

[13] Siegel SW, Richardson DA, Miller KL, Karram MM, Blackwood NB, Sand PK, et al. Pelvic floor electrical stimulation for the treatment of urge and mixed urinary incontinence in women. Urology. 1997;50:934-40. 10.1016/S0090-4295(97)00484-6 9426726

[14] Yamanishi T, Yasuda K, Sakakibara R, Hattori T, Suda S. Randomized, double-blind study of electrical stimulation for urinary incontinence due to detrusor overactivity. Urology. 2000;55:353-7. 10.1016/s0090-4295(99)00476-8 10699609

[15] Wang S, Lv J, Feng X, Lv T. Efficacy of Electrical Pudendal Nerve Stimulation versus Transvaginal Electrical Stimulation in Treating Female Idiopathic Urgency Urinary Incontinence. J Urol. 2017;197:1496-501. 10.1016/j.juro.2017.01.065 28153510

[16] Scaldazza CV, Morosetti C, Giampieretti R, Lorenzetti R, Baroni M. Percutaneous tibial nerve stimulation versus electrical stimulation with pelvic floor muscle training for overactive bladder syndrome in women: results of a randomized controlled study. Int Braz J Urol. 2017;43:121-6. 10.1590/S1677-5538.IBJU.2015.0719 PMC5293392 28124534

[17] Goode PS, Locher JL, Bryant RL, Roth DL, Burgio KL. Measurement of postvoid residual urine with portable transabdominal bladder ultrasound scanner and urethral catheterization. Int Urogynecol J Pelvic Floor Dysfunct. 2000;11:296-300. 10.1007/s001920070020 11052565

[18] Lee HE, Cho SY, Lee S, Kim M, Oh SJ. Short-term Effects of a Systematized Bladder Training Program for Idiopathic Overactive Bladder: A Prospective Study. Int Neurourol J. 2013;17:11-7. 10.5213/inj.2013.17.1.11 PMC3627992 23610706

[19] Berghmans B, van Waalwijk van Doorn E, Nieman F, de Bie R, van den Brandt P, et al. Efficacy of physical therapeutic modalities in women with proven bladder overactivity. Eur Urol. 2002;41:581-7. 10.1016/s0302-2838(02)00178-1 12074773

[20] Berghmans LC, Hendriks HJ, De Bie RA, van Waalwijk van Doorn ES, Bø K, et al. Conservative treatment of urge urinary incontinence in women: a systematic review of randomized clinical trials. BJU Int. 2000;85:254-63. 10.1046/j.1464-410x.2000.00434.x 10671878

[21] Abdelbary AM, El-Dessoukey AA, Massoud AM, Moussa AS, Zayed AS, Elsheikh MG, et al. Combined Vaginal Pelvic Floor Electrical Stimulation (PFS) and Local Vaginal Estrogen for Treatment of Overactive Bladder (OAB) in Perimenopausal Females. Randomized Controlled Trial (RCT). Urology. 2015;86:482-6. 10.1016/j.urology.2015.06.007 26135813

[22] Nygaard IE, Kreder KJ, Lepic MM, Fountain KA, Rhomberg AT. Efficacy of pelvic floor muscle exercises in women with stress, urge, and mixed urinary incontinence. Am J Obstet Gynecol. 1996;174(1 Pt 1):120-5. 10.1016/s0002-9378(96)70383-6 8571994

[23] O’Sullivan R, Karantanis E, Stevermuer TL, Allen W, Moore KH. Definition of mild, moderate and severe incontinence on the 24-hour pad test. BJOG. 2004;111:859-62. 10.1111/j.1471-0528.2004.00211.x 15270937

[24] Rahmani N, Mohseni-Bandpei MA. Application of perineometer in the assessment of pelvic floor muscle strength and endurance: a reliability study. J Bodyw Mov Ther. 2011;15:209-14. 10.1016/j.jbmt.2009.07.007 21419362

[25] Tarcan T, Mangır N, Özgür MÖ, Akbal C. OAB-V8 Overactive Bladder Questionnaire Validation Study. (Turkish) Üroloji Bülteni 2012;21:113-6. [Internet] Available at. <https://kontinansdernegi.org/wp-content/uploads/2021/08/oab-v8-asiri-aktif-mesane-sorgulama-formu.pdf>

[26] Acquadro C, Kopp Z, Coyne KS, Corcos J, Tubaro A, Choo MS, et al. Translating overactive bladder questionnaires in 14 languages. Urology. 2006;67:536-40. Erratum in: Urology. 2007;69:202. Oh, Seung June [added]. 10.1016/j.urology.2005.09.035 16527574

[27] Cam C, Sakalli M, Ay P, Cam M, Karateke A. Validation of the short forms of the incontinence impact questionnaire (IIQ-7) and the urogenital distress inventory (UDI-6) in a Turkish population. Neurourol Urodyn. 2007;26:129-33. 10.1002/nau.20292 17083117

